# Prognostic value of ferritin in ASCT MM patients: integration with GEP models and ISS series systems

**DOI:** 10.1038/s41408-024-00998-9

**Published:** 2024-02-14

**Authors:** Wancheng Guo, Yihao Zhan, David Mery, Eric R. Siegel, Fumou Sun, Yan Cheng, Timothy Cody Ashby, Zijun Zhang, Clyde Bailey, Daisy V. Alapat, Hongling Peng, Samer Al Hadidi, Sharmilan Thanendrarajan, Carolina Schinke, Maurizio Zangari, Frits van Rhee, Guido Tricot, John D. Shaughnessy, Fenghuang Zhan

**Affiliations:** 1 Myeloma Center, Winthrop P. Rockefeller Cancer Institute, Department of Internal Medicine, University of Arkansas for Medical Sciences, Little Rock, AR USA; 2 Department of Haematology, Second Xiang-ya Hospital, Central South University, Changsha, Hunan China; 3 Department of Biostatistics, University of Arkansas for Medical Sciences, Little Rock, AR USA; 4 Department of Biomedical Informatics, University of Arkansas for Medical Sciences, Little Rock, AR USA; 5 Department of Pathology Clinical, University of Arkansas for Medical Sciences, Little Rock, AR USA

**Keywords:** Haematological cancer, Cancer

Dear Editor

Since the 1960s, various multiple myeloma (MM) risk factors have been identified, including bone lytic lesions, serum calcium, hemoglobin, and serum creatinine [1]. In attempts to predict tumor burden and prognosis, several MM staging systems were developed, such as the Durie/Salmon (DS) system [2], as well as other stage systems [3–5]. Beta-2-microglobulin (B2M) and C-reactive Protein (CRP) have been identified as strong risk factors [6, 7]. Other effective prognostic factors, such as lactate dehydrogenase (LDH) and aspartate aminotransferase (AST) have also been identified [8, 9]. Using commonly available clinical parameters, the International Staging System (ISS) was developed, which is based on B2M and albumin (ALB) for staging [10]. Conventional cytogenetics techniques, such as metaphase karyotyping and TriFISH provide chromosomal abnormality information relevant to MM prognosis [11–13]. Consequently, the Revised International Staging System (R-ISS) and Second Revision of the International Staging System (R2-ISS) were proposed [14, 15]. In the early 21st century, gene expression profiling (GEP) became more widely available to explore disease-specific molecular signatures, including MM. Using specific gene expression signatures, several myeloma scoring systems were developed, such as GEP70, GEP80, proliferation index (PI), and Sky92. These systems aided in identifying the approximately 20% of myeloma cases with the worst prognosis. The development of whole-genome sequencing technology has revealed unfavorable prognostic mutations in myeloma.

Here, we collected clinical variables, including gender, age, race, B2M, ALB, LDH, serum ferritin levels, et al. from 3446 consecutive MM patients diagnosed between 1983 and 2019, who had received autologous stem cell transplantation therapy and had serum ferritin testing in the University of Arkansas for Medical Sciences (UAMS). 90% had upfront transplantation (before disease progression) and 10% had a salvage transplant. Of the 3446 patients with MM, 1700 had GEP data, and more than 900 patients had TriFISH results (including 1q+ and 17p del). High serum ferritin was defined as ≥ 336 mg/L for males and ≥ 306 mg/L for females according to UAMS clinical standard of Ferritin high. Figure S1 presents the workflow for selecting patients’ data for analysis and subsets of these 3446 MM cases. Upfront transplantation was defined as a transplant before any relapse, while a salvage transplant was defined as a transplant after relapse. Subgroups in this paper were summarized in Table S1. The collection of all data was approved by the Institutional Review Board of the UAMS and written informed consent was obtained from all subjects for the procurement of samples, following the guidelines outlined in the Declaration of Helsinki.

Among the 3446 ASCT patients with ferritin levels available, 2051 (60%) were male and 1395 (40%) were female. The median age at the time of first chemotherapy was 59 years. As shown in Table S2, most patients were Caucasian (85%) or African American (12%). ISS stage information was available for 3408 (99%) of these patients, with 1506 (44%) classified as ISS stage I, 1025 (30%) as ISS stage II, and 877 (25%) as ISS stage III. There were 2432 (71%) patients who experienced disease progression and 2148 (62%) deaths. The 5-year progression-free survival rate for ASCT patients was 51%, and the 5-year overall survival rate was 67%. We assessed the prognostic significance of serum ferritin across various demographic subgroups. Univariable and multivariable cox analyses were performed in those demographic and clinical serum indexes (Table S3, 4), indicating that age, serum ferritin level, ALB, B2M, LDH, Creatinine and ISS stage are prognostic factor for MM patients’ overall survival (OS) and progression-free survival (PFS). As shown in Fig. 1, Figure S2, serum ferritin emerged as a prognostic biomarker for MM patients, irrespective of gender, race, or age. An elevated ferritin level is associated with a poorer prognosis in patients (Figure S3). Additionally, we tested the performance of ferritin in four effective GEP models (GEP70, GEP80, proliferation index, and Sky92), which identify 10–23% patients with the worst outcome (Figure S4A, C, E, G). We combined ferritin high/normal with these GEP models. The high-risk and low-risk groups predicted by GEP were significantly separated according to the ferritin level (Figure S4B, D, F, H). Similar results were observed for overall survival (Figure S5). A multivariate cox regression test was performed using gender, age, serum ferritin, and four GEP scores from 1688 patients (Tables S5, S6), indicating that ferritin remained an independent risk factor for both PFS (HR: 1.67, 95%CI: [1.36, 2.05]) and OS (HR: 1.68, 95%CI: [1.48, 1.92]).

**Fig. 1:**
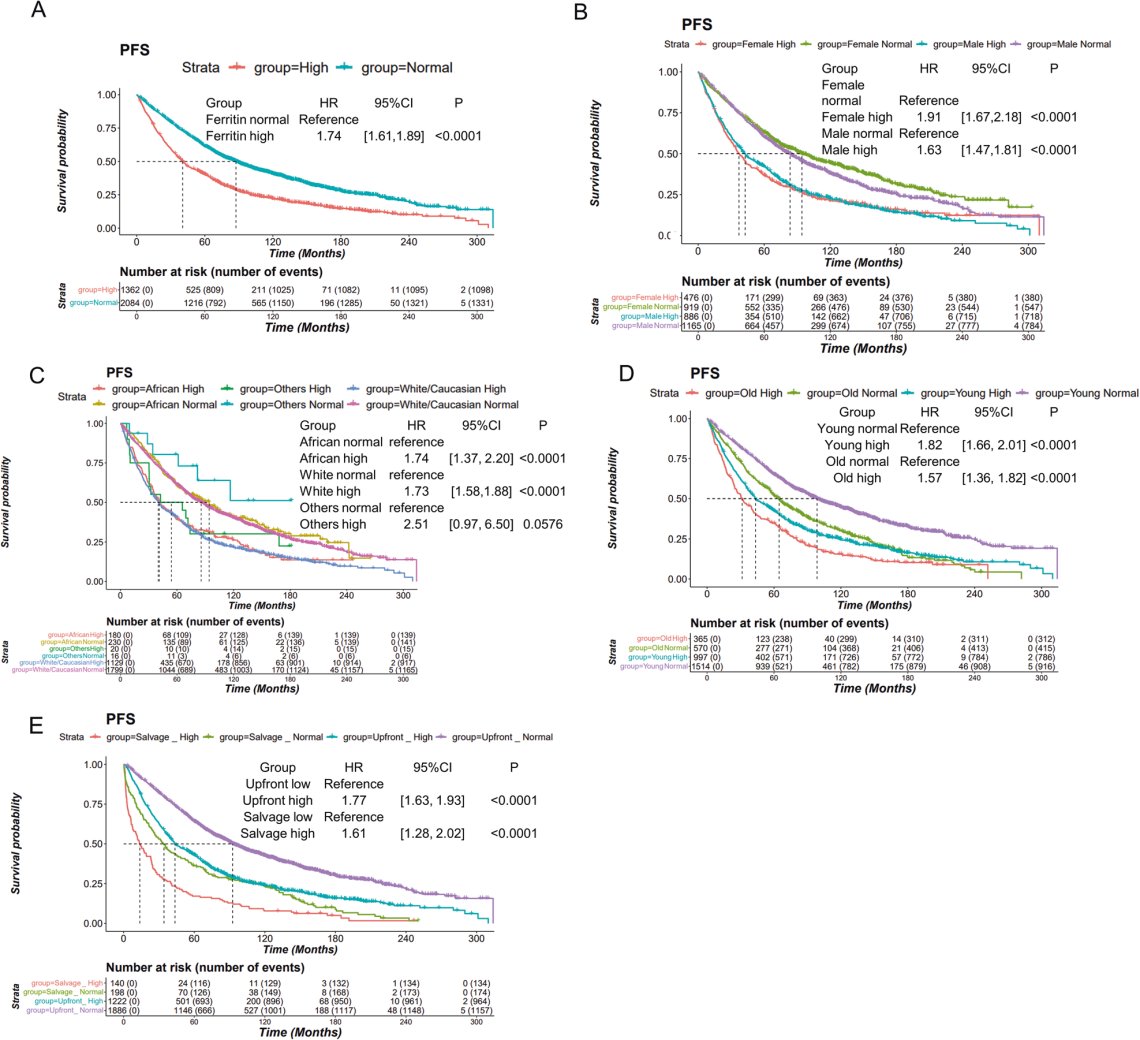
PFS of ferritin and ferritin in different age, gender, race subgroups. **A** Progression-free survival curves of ferritin high/normal group. **B** Progression-free survival curves of ferritin high/normal in male and female ASCT MM. **C** Progression. free survival curves of ferritin high/normal in White, African and others. **D** Progression-free survival curves of ferritin high/normal in the young (<65) and old. **E** Progression-free survival curves of ferritin high/normal in MM patients who received ASCT upfront versus as salvage therapy.

We conducted further analysis to evaluate the prognostic impact of ferritin in patients with different stages of the ISS system. Of the 3446 patients, 3408 had information on their ISS stage. The ISS system is a widely used, simple, and effective model for MM staging. The results showed that patients in ISS stage II had a hazard ratio of 1.20 (95%CI: [1.09, 1.32], for PFS) and patients in ISS stage III had a hazard ratio of 1.65 (95%CI: [1.50, 1.82], for PFS), compared to patients in ISS stage I (Fig. 2A). We combined the ISS staging system with high/normal ferritin levels. In all ISS stages, patients with high ferritin had a significantly worse OS and PFS (Fig. 2B, Figure S6B). Furthermore, a multivariable cox-regression analysis in 3329 patients with complete data indicated that ferritin remained an independent risk factor for MM prognosis, even after adjusting for gender, age, creatine, LDH, ISS stage system, and parameters used for the ISS stage construction (Table S7, Table S8).

**Fig. 2:**
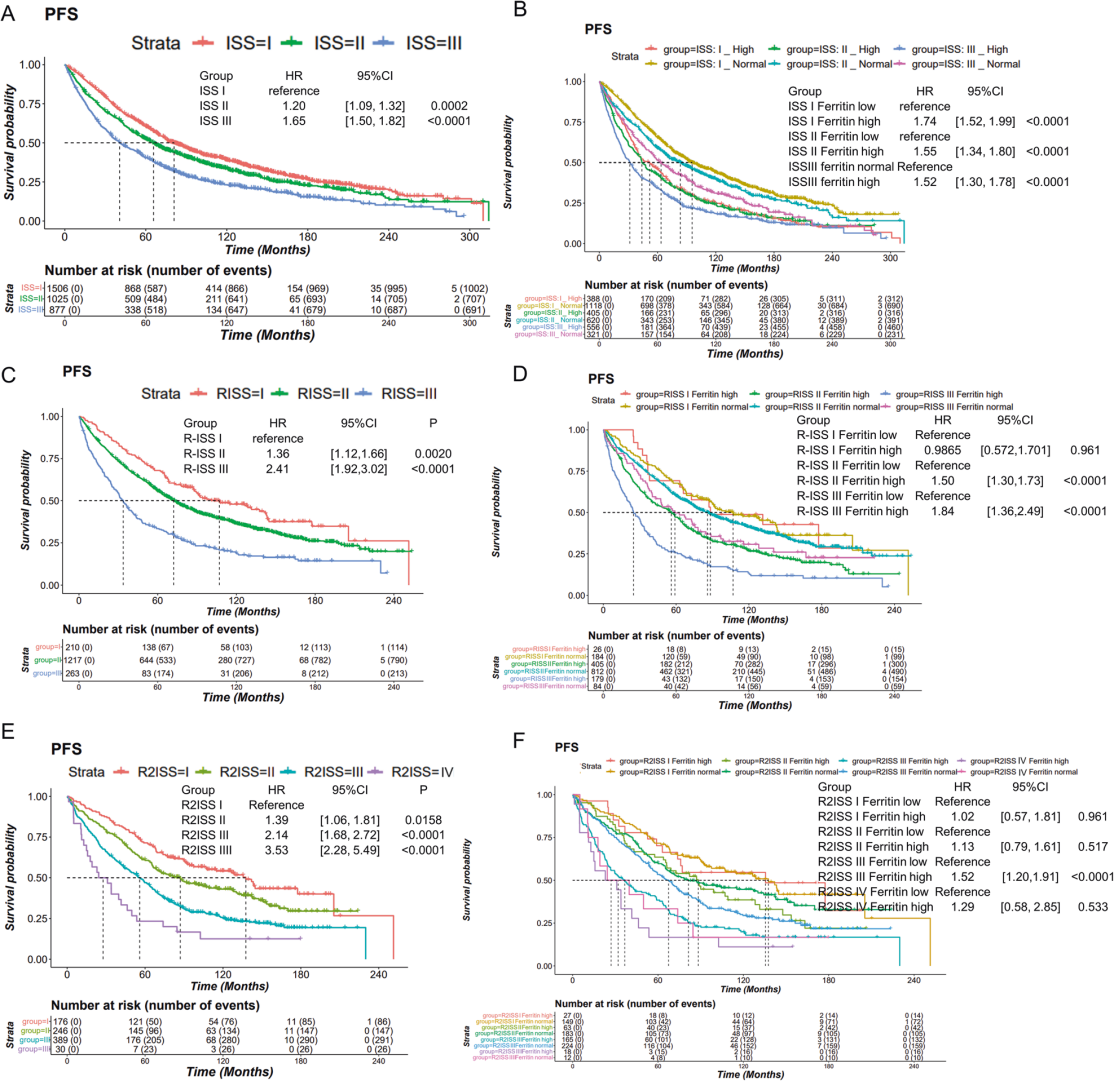
Ferritin’s progression-free survival prognostic effect in different ISS series systems’ subgroups. **A** Progression-free survival curves of different ISS stages in ASCT MM. **B** Progression-free survival curves of ferritin high/normal in three ISS stages. **C** Progression-free survival curves of different R-ISS stages in ASCT MM. **D** Progression-free survival curves of ferritin high/normal in three R-ISS stages. **E** Progression-free survival curves of different R2-ISS stages in ASCT MM. **F** Progression-free survival curves of ferritin high/normal in four R2-ISS stages.

The R-ISS and R2-ISS systems, assessing MM prognosis using serum tests and TriFISH, were developed in 2015 and 2022 [14, 15], respectively. We identified a subset of 1690 patients with both R-ISS stage and ferritin, and a subset of 841 patients with both R2-ISS and ferritin. Both R-ISS and R2-ISS systems demonstrated improved separation survival curves of patients which impacted outcomes (Fig. 2C, E, Figure S6C, E). Ferritin significantly influenced outcomes of patients in R-ISS stage II and III, but not stage I (Fig. 2D, Figure S6D). For the R2-ISS system, ferritin had a significant impact on the prognosis of patients in R2-ISS stage III and a potential impact for R2-ISS stage IV (Fig. 2F, Figure S6F). The sample size of the R2-ISS stage IV subset (*n* = 30) was very small, which most likely explains the lack of statistical significance. In addition, the proportion of patients with high ferritin levels increased following in higher R-ISS and R2-ISS stages (Table S9), indicating that high ferritin levels were more common in high-risk MM patients. The median overall survival of patients with high/low ferritin level was shown in Table S10, showing that high ferritin significantly impacted survival predicted by ISS-series stages. A multivariable cox-regression analysis was performed using gender, age, and parameters for constructing R-ISS and R2-ISS from 841 MM patients, demonstrating that ferritin consistently remained an independent risk factor for both PFS and OS (OS: HR: 1.36, 95% CI: [1.11, 1.68]; PFS: HR: 1.23, 95% CI: [1.02, 1.48]). For non-transplant MM patients, we analyzed 708 MM cases and found that serum ferritin showed a similar result as a high-risk factor in MM prognosis (Figure S7,8 and Table S11).

### Supplementary information

Supplementary methods and results

## Data Availability

For original data, please contact FZhan@uams.edu.
