# Supplementary material for: Prognostic value of ferritin in ASCT MM patients: integration with GEP models and ISS series systems
Source: Blood Cancer J. 2024 Feb 14;14(1):30. doi: 10.1038/s41408-024-00998-9 (PMC10866867; doi:10.1038/s41408-024-00998-9)
Supplement: Supplementary file 1 — Supplementary methods and results [file 41408_2024_998_MOESM1_ESM.docx]

Prognostic Value of Ferritin in ASCT MM patients: Integration with GEP Models and ISS Series Systems

Data supplement

Catalog

[1. Supplementary methods 2](#_Toc155165846)

[Patient population 2](#_Toc155165847)

[Calculation of GEP scores and chromosome translocation prediction 2](#_Toc155165848)

[Fluorescence in situ hybridization 3](#_Toc155165849)

[Study end points 3](#_Toc155165850)

[Statistical analyses 4](#_Toc155165851)

[2. Supplement results 5](#_Toc155165852)

[Supplementary figure1. Flow chart of patients screening 5](#_Toc155165853)

[Supplementary figure2. Overall survival curves of ferritin in different subgroups of age, gender, race, and ASCT type. 6](#_Toc155165854)

[Supplementary figure3 Survival curves of ferritin based on quartile and decile grouping. 7](#_Toc155165855)

[Supplementary figure4 Progression-free survival curves of ferritin and GEP models 8](#_Toc155165856)

[Supplementary figure5 Overall survival curves of ferritin and GEP models 9](#_Toc155165857)

[Supplementary figure6 Overall survival curves of ISS series systems and ferritin 10](#_Toc155165858)

[Supplementary figure7 Progression-free survival curves of ferritin high/normal non-transplant MM. 11](#_Toc155165859)

[Supplementary figure8 Overall survival curves of ferritin high/normal non-transplant MM. 12](#_Toc155165860)

[Supplementary table1. Summary table of subsets in this paper. 12](#_Toc155165861)

[Supplementary table2. Baseline information of 3446 ASCT MM patients 13](#_Toc155165862)

[Supplementary table3. Univariable Cox regressions of progression-free survival on demographic and serum-index variables 15](#_Toc155165863)

[Supplementary table4. Univariable Cox regressions of overall survival on demographic and serum-index variables 16](#_Toc155165864)

[Supplementary table5. Multivariable Cox of ferritin and GEP scores (Progression-free survival) 17](#_Toc155165865)

[Supplementary table6. Multivariable Cox of ferritin and GEP scores (Overall survival) 18](#_Toc155165866)

[Supplementary table7 Multivariable Cox regressions of progression-free survival on demographic and serum-index variables in the 3329-MM subset 18](#_Toc155165867)

[Supplementary table8. Multivariable Cox regressions of overall survival on demographic and serum-index variables in the 3329-MM subset 19](#_Toc155165868)

[Supplementary table9. Percentage of high-ferritin MM patients in ISS-series stages 20](#_Toc155165869)

[Supplementary table10. Median survival of stages in ISS series systems combine with ferritin 20](#_Toc155165870)

[Supplementary table11 Baseline of 708 non-transplant MM patients 21](#_Toc155165871)

[Reference 22](#_Toc155165872)

# 1. Supplementary methods

## Patient population

The data for this study were collected from 3446 consecutive MM patients diagnosed between 1983 and 2019, who had received autologous stem cell transplantation therapy and had serum ferritin testing in the University of Arkansas for Medical Sciences (UAMS). Figure S1 presents the workflow for selecting patients' data for analysis and subsets of the 3446 MM cases. Upfront transplantation was defined as a transplant before any relapse, while a salvage transplant was defined as a transplant after relapse.

We collected various parameters, including gender, age, race, B2M, ALB, LDH, and serum ferritin levels before transplantation. 90% had upfront transplantation (before disease progression) and 10% had a salvage transplant. Among the 3446 MM patients, 1700 had GEP data, and more than 900 patients had TriFISH results (including 1q+ and 17p del). High serum ferritin was defined as ≥ 336mg/L for males and ≥ 306mg/L for females according to UAMS clinical standard of Ferritin high. Subgroups in this paper were summarized in Table S1. The collection of all data was approved by the Institutional Review Board of the University of Arkansas for Medical Sciences, and written informed consent was obtained from all subjects for the procurement of samples, following the guidelines outlined in the Declaration of Helsinki.

## Calculation of GEP scores and chromosome translocation prediction

Bone marrow samples were obtained under local anesthesia from the posterior iliac crest. Plasma cell isolation was performed by immunomagnetic bead selection using monoclonal mouse anti-human CD138 antibody by the AutoMACS automated separation system^1^. We selected those samples with a post-sorting purity more than 80%. Then, RNA was extracted, and gene expression profiling was performed. Plasma cell purifications and gene expression profiling (GEP) were conducted using the Affymetrix U133Plus2.0 microarray (Santa Clara, CA), following the previously established protocols^1, 2^.

The GEP70^3^, GEP80^4^, proliferation index and Sky92^5^ scores of these patients were calculated. The t(4;14) and t(14;16) chromosome translocations were based on GEP expression “Spikes” for the *FGFR3* and/or *NSD2* ^1^ and *c-MAF* ^2^, respectively as well as classification in either the MS or MF molecular subgroups as reported by Zhan et al., 2006.

## Fluorescence in situ hybridization

Erythrocytes were removed from bone marrow aspirates by Ficoll-Hypaque gradient-centrifugation separation. For 17p FISH, to identify TP53 deletions, a SpectrumRed-labeled DNA probe (LSI p53; Vysis, Downers Grove, IL) was utilized along with a SpectrumGreen-labeled probe (CEP17, Vysis) targeting the α-satellite DNA centromere of chromosome 17. For 1q gain, bacterial artificial chromosomes (BACs) RP11-307C12 located at 1q21 and RP11-32D17 located at 1q31 were acquired from BAC/PAC Resources (Oakland, CA).

For 17p del, the interphase-FISH procedure employed for sample analysis has been previously described^6-8^. Based on FISH studies conducted on normal bone marrow mononuclear cells, the upper limit of normal plus three standard deviations (SD) for TP53 deletions were found to be less than 10%^9^. Therefore, we set the background cutoff level for the probe sets at 10%.

For 1q gain, the interphase-FISH procedure utilized in this study has been previously documented^6^. Cytospin preparations of mononuclear cells obtained from bone marrow aspirates were subjected to Ficoll separation and fixed with ethanol. The hybridization of probes, accompanied by AMCA-labeled antibodies targeting κ or λ immunoglobulin light chains (Vector Laboratories, Burlingame, CA), was performed. The resulting slides were stored at -20°C until FISH analyses were conducted. A minimum of 100 clonal plasma cells, predominantly 100 cells, were evaluated for interphase FISH signals in each patient. The presence of at least 3 copies in at least 20% of clonal plasma cells was considered as evidence of gain/amplification. To explore the impact of Amp1q21 on clinical outcomes and distinguish between different categories, Amp1q21 was classified into two groups: (1) 3 copies of 1q21 (with less than 20% of clonal plasma cells showing at least 4 copies) and (2) at least 4 copies of 1q21 (with at least 20% of clonal plasma cells showing at least 4 copies).

## Study end points

The primary study endpoint was progression free survival (PFS), subtracting the first chemotherapy date from first progression date/death date/last contact date (median PFS: 66.0 months). The secondary end point was overall survival (OS), subtracting the first chemotherapy date from death date/last contact date (median OS: 110.3 months).

Considering the medium follow-up time is 7·27 years, we selected the progression free survival as primary event endpoints. For those patients with progression events after 2006, the definition of disease progression refers to IMWG standard^10^ (<https://www.myeloma.org/resource-library/international-myeloma-working-group-imwg-uniform-response-criteria-multiple>). For those with progression events before IMWG standard, progression events were defined by doctors.

## Statistical analyses

In this study, only complete data was analyzed. Medians and inter-quartile ranges were used to describe continuous variables, while percentages were used for categorical variables. For continuous clinical variables (age, B2M, ALB, LDH, and ferritin), we transformed them into categorical variables based on clinical standards. GEP70, GEP80, PI, and Sky92 scores were defined as high/low using the original literature standards.

For clinical continuous variables (age, ferritin, LDH, ALB, B2M and creatinine), they were transformed into binary by clinical standard. For GEP scores (GEP70^3^, GEP80^4^, PI^2^ and SKY92^5^), they were transformed into binary as original paper described. Supplementary table2 and Table S7 show the exact border of the two groups.

Originally, we selected 3446 patients with clear PFS, OS, ferritin level and ISS level information. We found that some of the ALB and B2M values didn’t match the ISS stage. So, ISS stages were re-calculated using ALB and B2M level^11^. We also calculated the R-ISS^12^ stage and R2-ISS^13^ stage information based on the clinical and chromosome abnormality data.

We only analyze the complete data and evaluate ferritin’s prognostic role by taking subgroups. The main subgroups involved in this article are as followed

Kaplan-Meier analysis was utilized for calculating cumulative survival probability^14^, and the log-rank test^15^ was used to determine differences between Kaplan-Meier curves. Univariable and multivariable Cox regression analysis were used to evaluate the prognostic significance of each parameter.^16^

We used R4.0.5 software for statistical analysis. Survival (version 3.2-10) and survminer (version 0.4.9.999) were used for survival analysis and visualization. CBCgrps^17^ (version 2.8.2) was used for baseline table..

# 2. Supplement results

## Supplementary figure1. Flow chart of patients screening


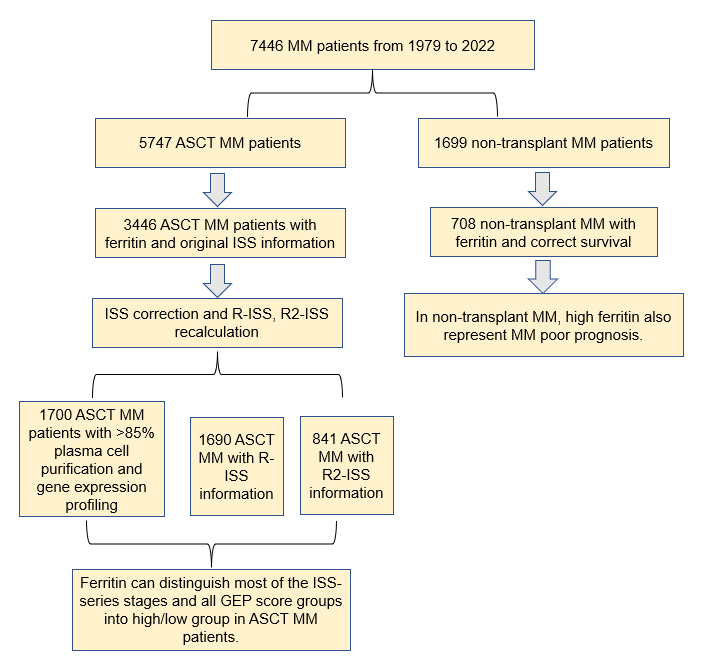


Abbreviations. MM, multiple myeloma; ASCT, autologous stem cell transplantation; ISS, the International Stage System; R-ISS, Revised version of the International Stage System; R2-ISS, Second Revision of the International Stage System; GEP, gene expression profiling. Notably, we corrected the ISS information using the ALB and B2M values, and finally got 3408 patients with corrected ISS information.

## Supplementary figure2. Overall survival curves of ferritin in different subgroups of age, gender, race, and ASCT type.


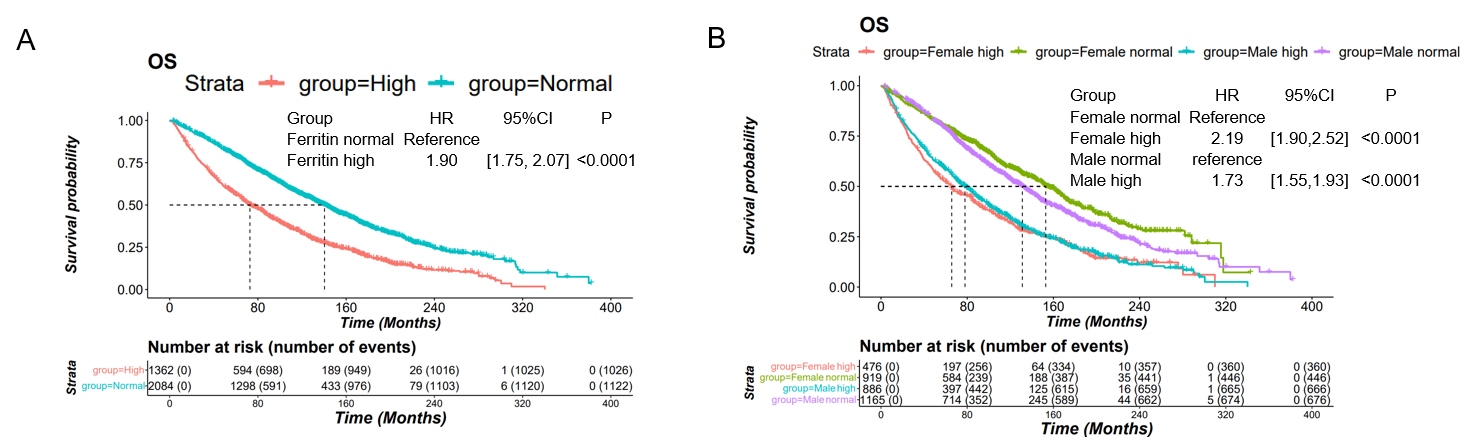


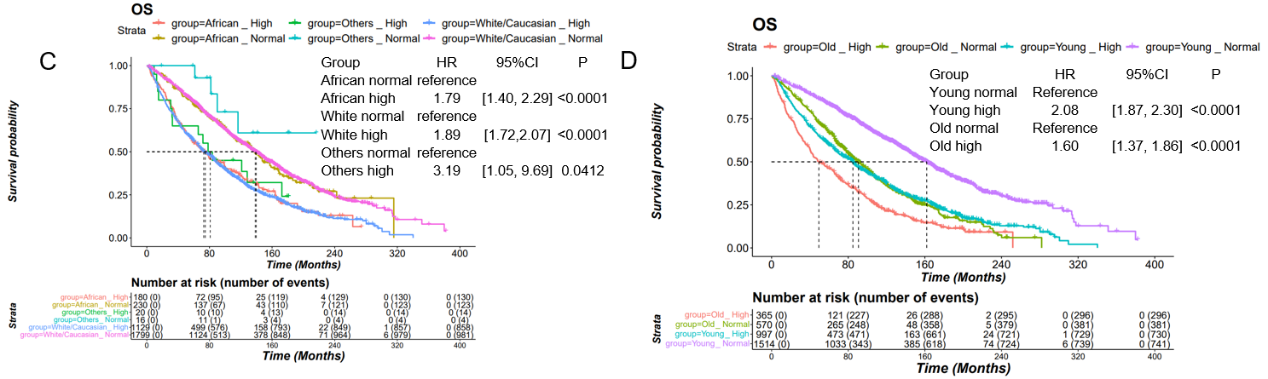


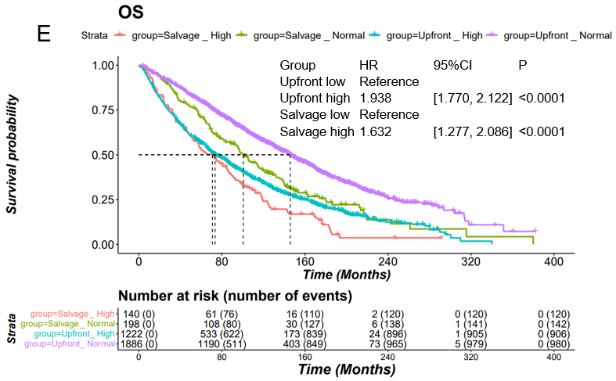


## Supplementary figure3 Survival curves of ferritin based on quartile and decile grouping.


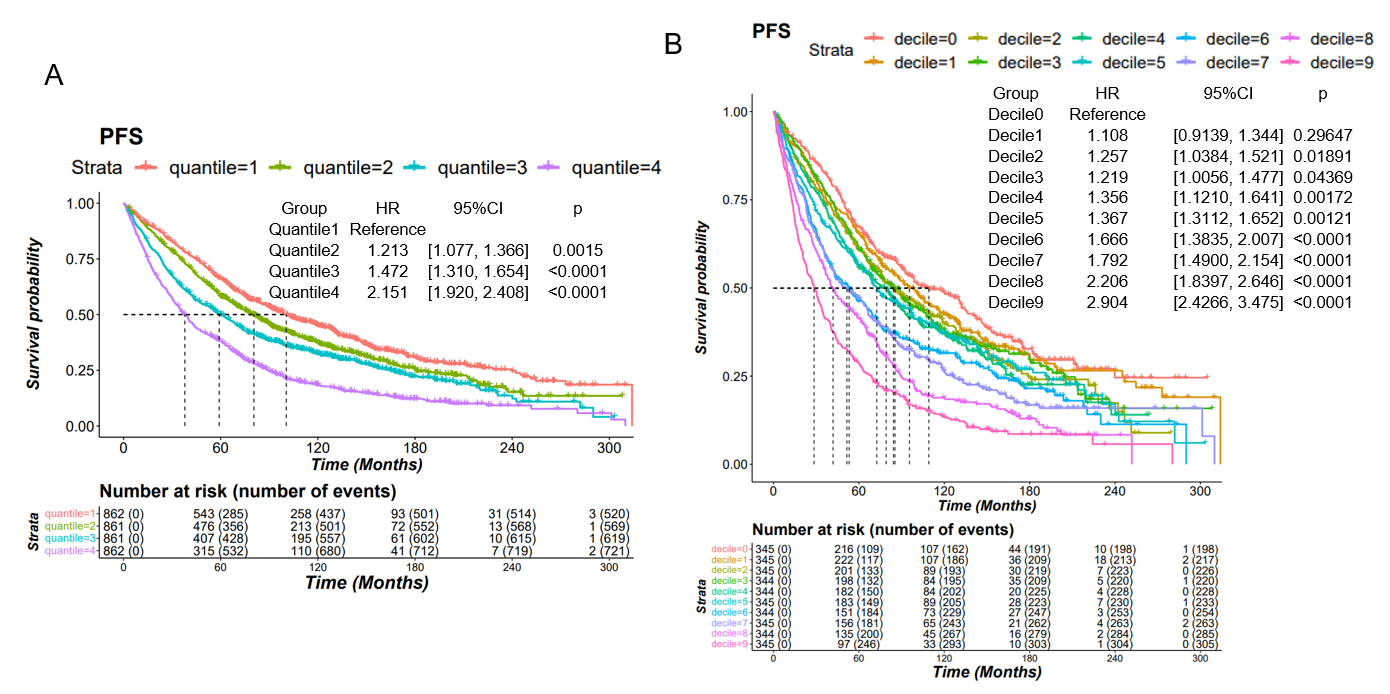


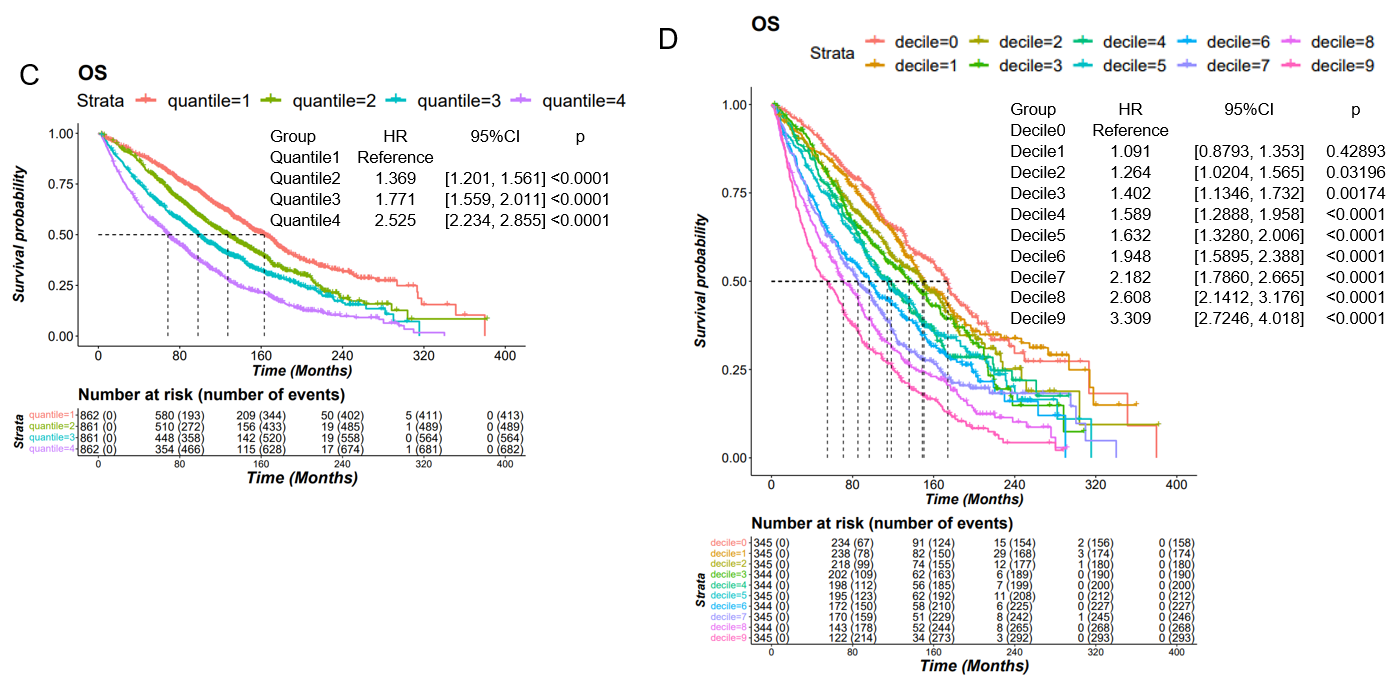


Abbreviation. PFS, progression free survival; OS, overall survival.

Group1-4 in figureS1 A, C represent the lowest ferritin to highest ferritin subset cut by quantile. Group1-10 in figureS1 B, D represent the lowest ferritin to highest ferritin subset cut by decile.

## Supplementary figure4 Progression-free survival curves of ferritin and GEP models


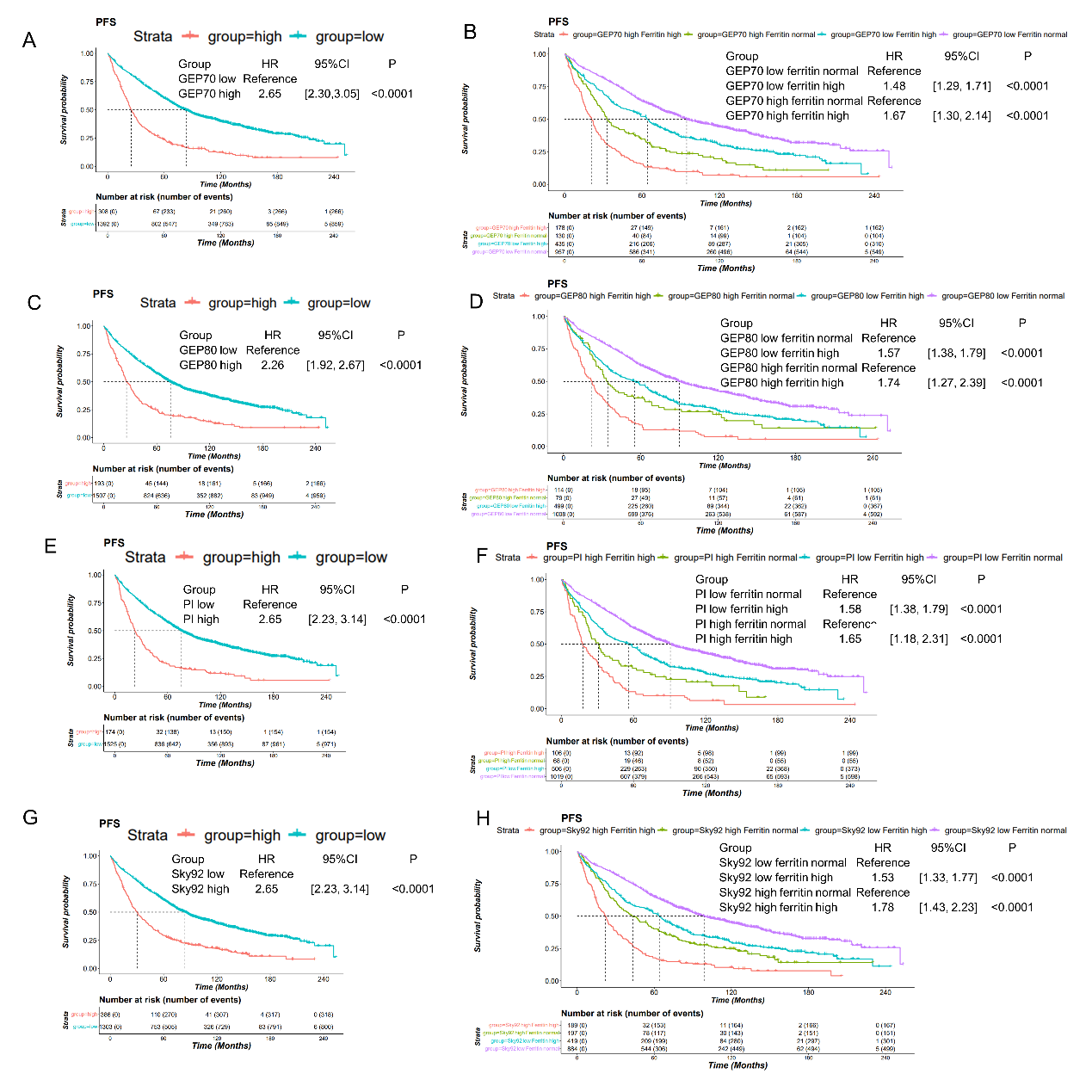


Abbreviation. GEP, gene expression profiling; PI, proliferation index; SKY92, skyline 92-gene signature

## Supplementary figure5 Overall survival curves of ferritin and GEP models


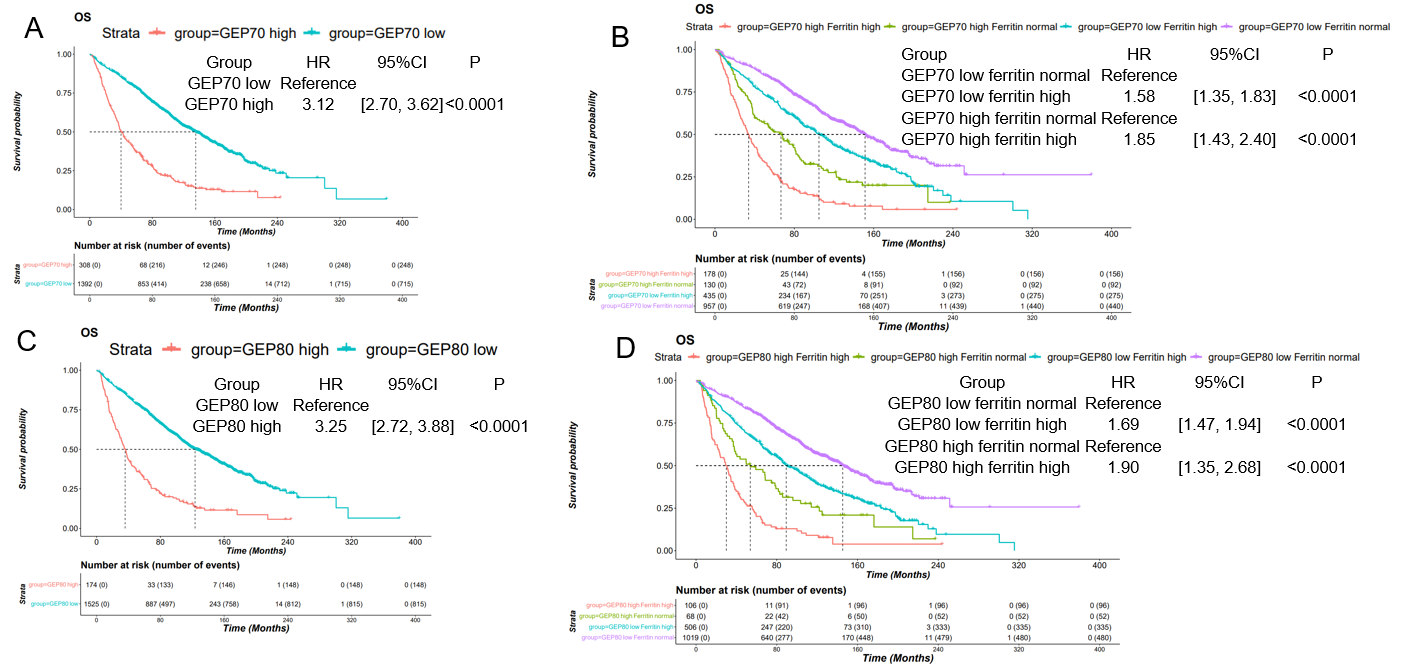


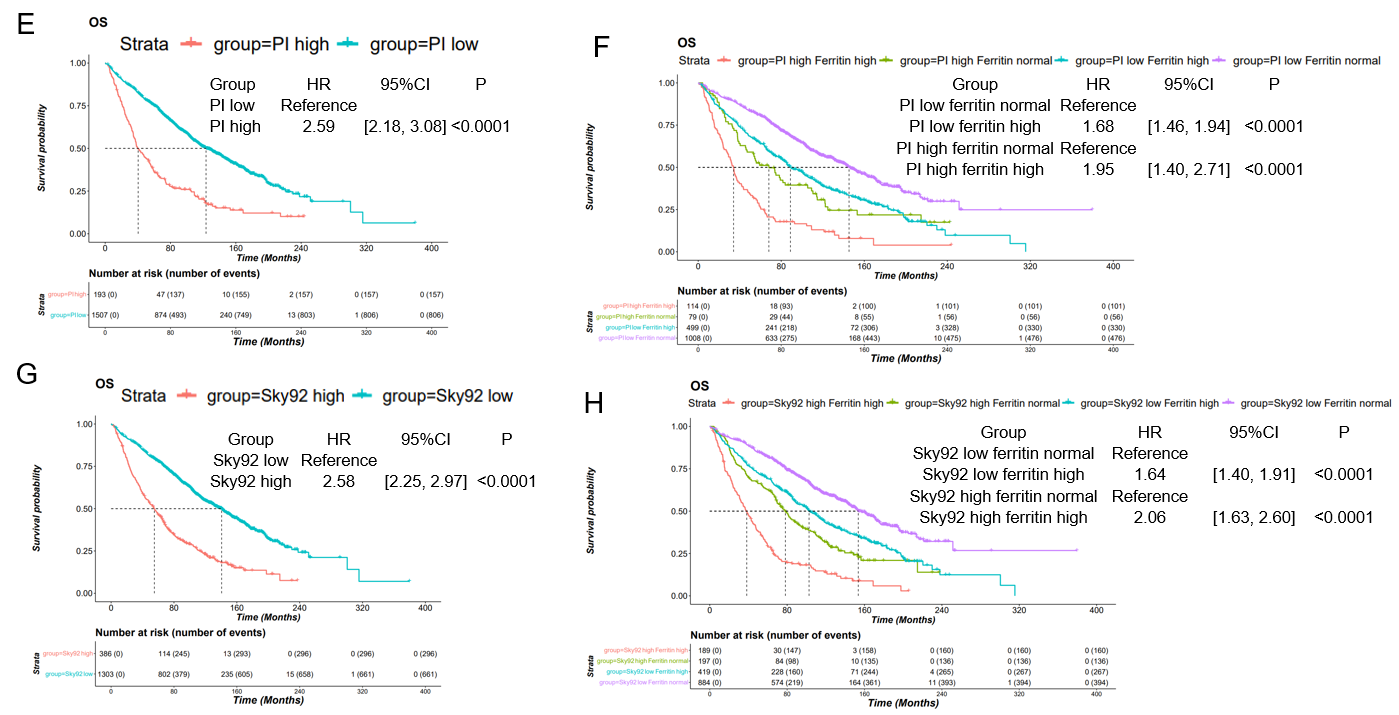


Abbreviation. GEP, gene expression profiling; PI, proliferation index; SKY92, skyline 92-gene signature

## Supplementary figure6 Overall survival curves of ISS series systems and ferritin


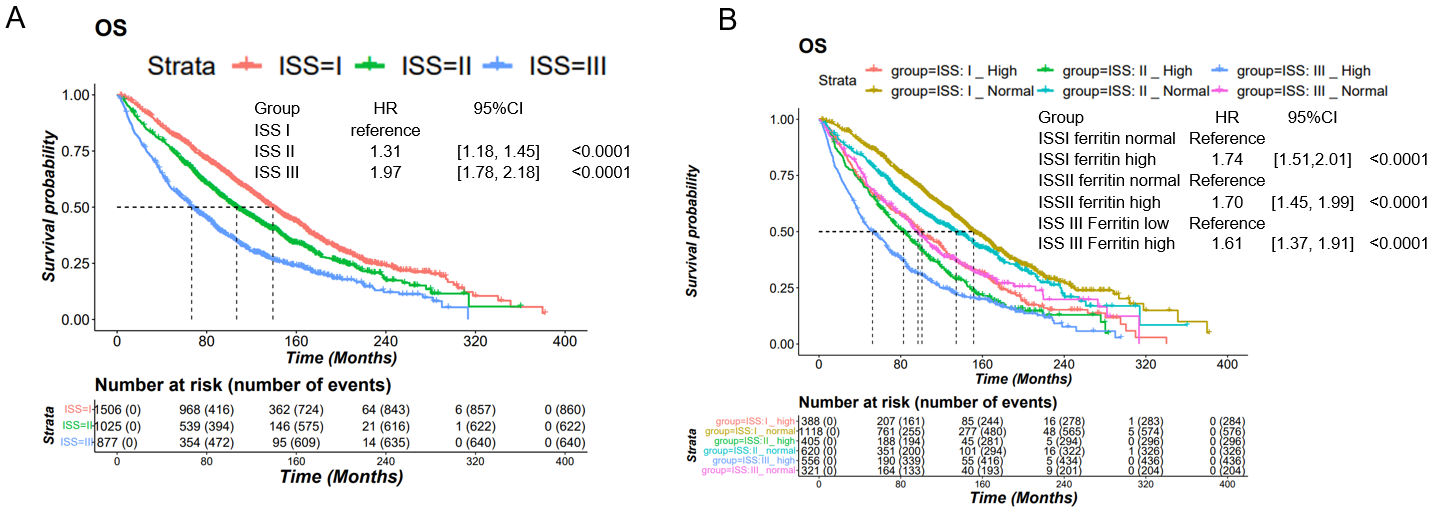


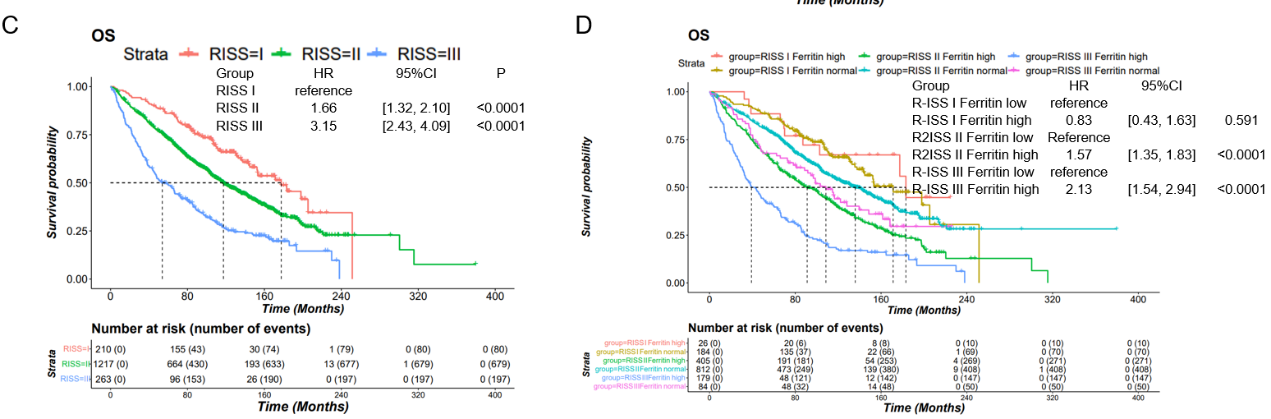


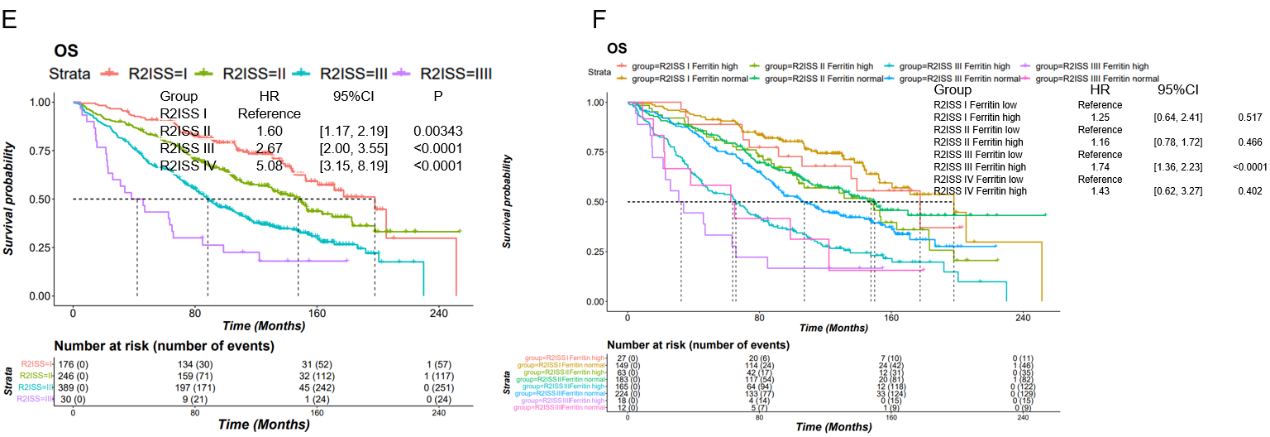


Abbreviation. R-ISS, Revised version of the International Stage System

A. Overall survival curves of ISS stage in transplant MM. B. Overall survival curves of ferritin high/normal in ISS stages. C. Overall survival curves of R-ISS stage in transplant MM. D. Overall survival curves of ferritin high/normal in R-ISS stages. E. Overall survival curves of R2ISS stage in transplant MM. F. Overall survival curves of ferritin high/normal in R2ISS stages.

## Supplementary figure7 Progression-free survival curves of ferritin high/normal non-transplant MM.


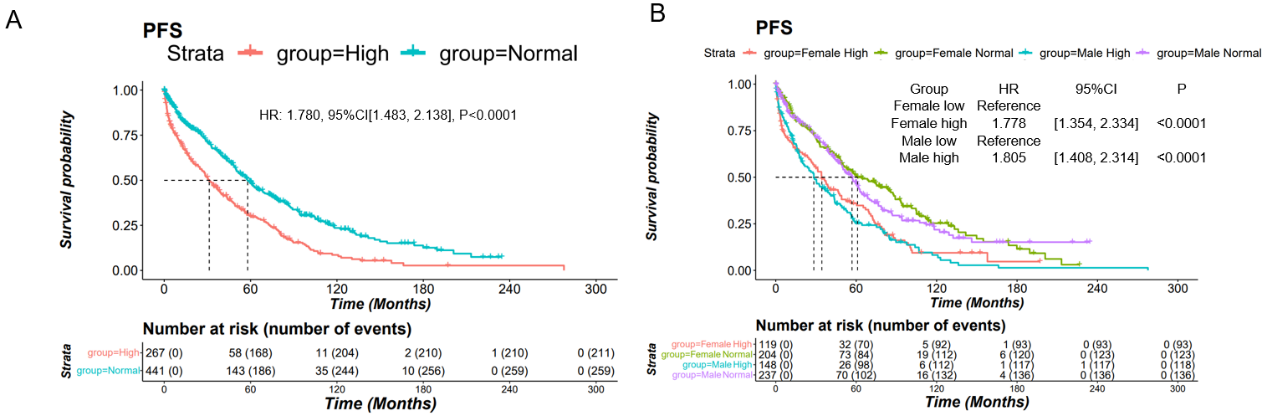


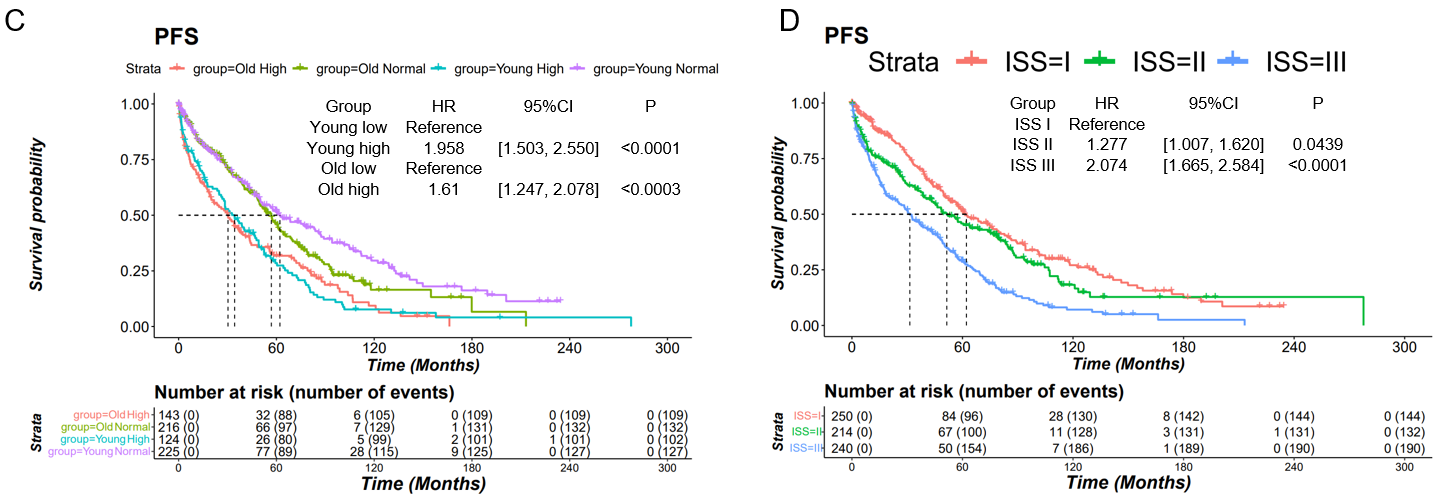


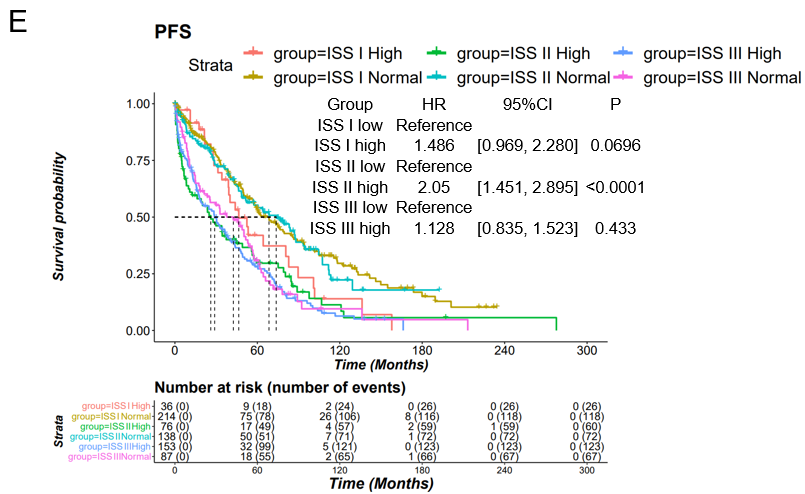


## Supplementary figure8 Overall survival curves of ferritin high/normal non-transplant MM.


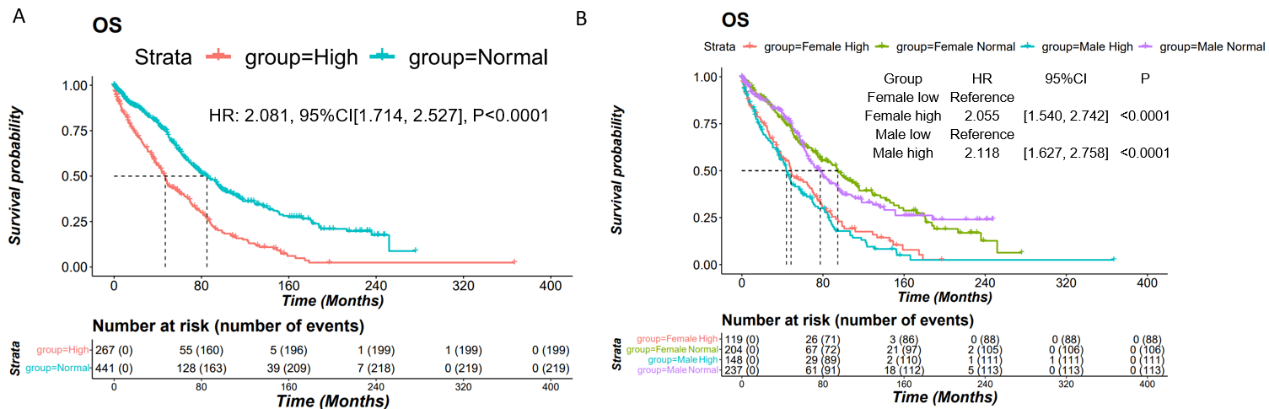


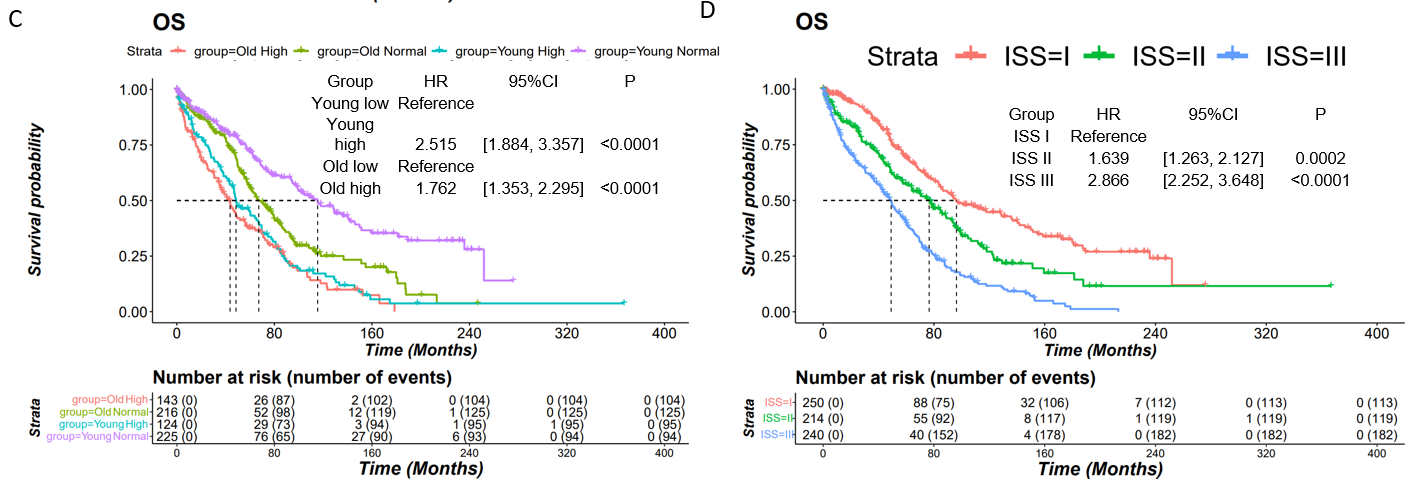


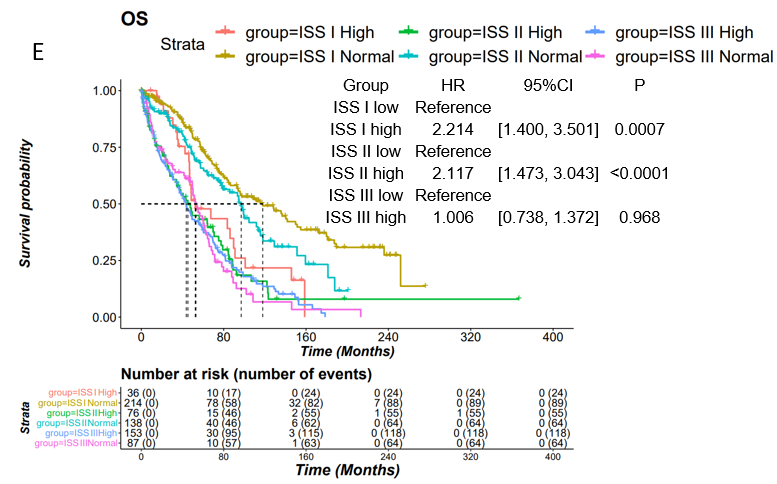


## Supplementary table1. Summary table of subsets in this paper.

| Subset | **Number of patients** |
| --- | --- |
| Complete ferritin and race information | 3374 |
| Complete ferritin and ISS information | 3408 |
| Complete ferritin and GEP score information | GEP70 and PI group 1700, GEP80 score group 1699, SKY92 score group 1689 |
| Complete ferritin and R-ISS information | 1690 |
| Complete ferritin and R2-ISS information | 841 |
| All information group | 815 |

Abbreviation. ISS, the International Stage System; R-ISS, Revised version of the International Stage System; R2-ISS, Second Revision of the International Stage System; GEP, gene expression profiling.

## Supplementary table2. Baseline information of 3446 ASCT MM patients

| Variables | Total (n = 3446) | Ferritin high (n = 1362) | Ferritin normal (n = 2084) | p |
| --- | --- | --- | --- | --- |
| Sex, n (%) |  |  |  | <0.001 |
| Female | 1395 (40) | 476 (35) | 919 (44) |  |
| Male | 2051 (60) | 886 (65) | 1165 (56) |  |
| Age Median (Q1, Q3) --yr. | 58.96 (51.53, 65.62) | 58.91 (51.7, 65.6) | 58.99 (51.45, 65.65) | 0.7 |
| Race, n (%) |  |  |  | 0.1 |
| Asian | 19 (1) | 9 (1) | 10 (0) |  |
| Black | 410 (12) | 180 (13) | 230 (11) |  |
| Native American | 14 (0) | 9 (1) | 5 (0) |  |
| Pacific Islander | 3 (0) | 2 (0) | 1 (0) |  |
| White / Caucasian | 2928 (85) | 1129 (83) | 1799 (86) |  |
| NA | 72 (2) | 33 (2) | 39 (2) |  |
| Serum Ferritin, Median (Q1, Q3) --mg/L | 235.65 (99.15, 523.3) | 641.7 (449.68, 1095.17) | 120.05 (62.2, 201.62) | <0.001 |
| Serum ALB, Median (Q1, Q3) --g/dL | 3.9 (3.5, 4.3) | 3.8 (3.3, 4.2) | 4 (3.6, 4.3) | <0.001 |
| Serum B2M, Median (Q1, Q3) --mg/L | 3.3 (2.2, 5.6) | 4.5 (2.8, 8.83) | 2.8 (2.04, 4.3) | <0.001 |
| Serum LDH, Median (Q1, Q3) --IU/L | 160 (131, 201) | 173 (137.5, 232.5) | 153 (127, 187) | <0.001 |
| Serum Creatinine, Median (Q1, Q3) --mg/dL | 1 (0.8, 1.4) | 1.1 (0.9, 1.9) | 1 (0.8, 1.2) | <0.001 |
| ISS, n (%) |  |  |  | <0.001 |
| I | 1506 (44) | 388 (28) | 1118 (54) |  |
| II | 1025 (30) | 405 (30) | 620 (30) |  |
| III | 877 (25) | 556 (41) | 321 (15) |  |
| Unknow | 38 (1) | 13 (1) | 25 (1) |  |
| ASCT type |  |  |  | 0.489 |
| Salvage | 338 (10) | 140 (10) | 198 (10) |  |
| Upfront | 3108 (90) | 1222 (90) | 1886 (10) |  |
| PFS, n (%) |  |  |  | <0.001 |
| No-progressed/Missing | 1014 (29) | 262 (19) | 752 (36) |  |
| Progressed | 2432 (71) | 1100 (81) | 1332 (64) |  |
| PFS.TIME, Median (Q1, Q3) --mth | 61.17 (24.94, 112.19) | 39.98 (15.89, 86.96) | 72.7 (34.81, 125.23) | <0.001 |
| OS, n (%) |  |  |  | <0.001 |
| Alive/Missing | 1298(38) | 336(25) | 962(46) |  |
| Death | 2148(62) | 1026(75) | 1122(54) |  |
| OS. TIME, Median (Q1, Q3) --mth | 88.43(44.68, 139.15) | 66.95(28.34, 119.43) | 100.47(59.95, 149.25) | <0.001 |

## Supplementary table3. Univariable Cox regressions of progression-free survival on demographic and serum-index variables

| **Variables** | **Hazard ratio** | **95%CI** | **p-value** |
| --- | --- | --- | --- |
| Gender |  |  |  |
| Female | reference |  |  |
| Male | 1.13 | [1.04, 1.23] | 0.004 |
| Race |  |  | 0.51 |
| White/Caucasian | reference |  |  |
| African | 0.97 | [0.86, 1.10] | 0.63 |
| Others | 0.80 | [0.52, 1.23] | 0.30 |
| Age |  |  |  |
| Young (<65) | reference |  |  |
| Old | 1.44 | [1.32, 1.57] | <0.0001 |
| ISS stage |  |  | <0.0001 |
| ISS I | reference |  |  |
| ISS II | 1.20 | [1.09, 1.32] | 0.0002 |
| ISS III | 1.65 | [1.50, 1.82] | <0.0001 |
| Ferritin level |  |  |  |
| Normal | reference |  |  |
| High  (female>306, male>336) | 1.74 | [1.61, 1.89] | <0.0001 |
| ALB |  |  |  |
| Normal | reference |  |  |
| Low (<3.5) | 1.29 | [1.18, 1.41] | <0.0001 |
| B2M |  |  |  |
| Normal (<4) | reference |  |  |
| High | 1.46 | [1.34, 1.58] | <0.0001 |
| LDH |  |  |  |
| Normal (<190) | reference |  |  |
| High | 1.36 | [1.24, 1.48] | <0.0001 |
| Creatinine |  |  |  |
| Normal (<2) | reference |  |  |
| High | 1.35 | [1.21, 1.51] | <0.0001 |

Abbreviation. ISS, the International Stage System; ALB, Albumin; B2M, Beta-2 Microglobulin; LDH, Lactate Dehydrogenase.

## Supplementary table4. Univariable Cox regressions of overall survival on demographic and serum-index variables

| **Variables** | **Hazard ratio** | **95%CI** | **pval** |
| --- | --- | --- | --- |
| Gender |  |  |  |
| Female | reference |  |  |
| Male | 1.14 | [1.04, 1.24] | 0.003 |
| Race |  |  | 0.67 |
| White/Caucasian | reference |  |  |
| African | 1.03 | [0.9, 1.17] | 0.71 |
| Others | 0.83 | [0.52, 1.32] | 0.44 |
| Age |  |  |  |
| Young (<65) | reference |  |  |
| Old | 1.78 | [1.63, 1.95] | <0.0001 |
| ISS stage |  |  | <0.0001 |
| ISS I | reference |  |  |
| ISS II | 1.31 | [1.18, 1.45] | <0.0001 |
| ISS III | 1.97 | [1.78, 2.18] | <0.0001 |
| Ferritin level |  |  |  |
| Normal | reference |  |  |
| High  (female>306, male>336) | 1.90 | [1.75, 2.07] | <0.0001 |
| ALB |  |  |  |
| Normal | reference |  |  |
| Low (<3.5) | 1.45 | [1.31, 1.59] | <0.0001 |
| B2M |  |  |  |
| Normal (<4) | reference |  |  |
| High | 1.67 | [1.53, 1.82] | <0.0001 |
| LDH |  |  |  |
| Normal (<190) | reference |  |  |
| High | 1.51 | [1.38, 1.65] | <0.0001 |
| Creatinine |  |  |  |
| Normal (<2) | reference |  |  |
| High | 1.63 | [1.46, 1.83] | <0.0001 |

Abbreviation. ISS, the International Stage System; ALB, Albumin; B2M, Beta-2 Microglobulin; LDH, Lactate Dehydrogenase.

## Supplementary table5. Multivariable Cox of ferritin and GEP scores (Progression-free survival)

| **Variables** | **HR** | **95%CI** | **P value** |
| --- | --- | --- | --- |
| Gender |  |  |  |
| Female | Reference |  |  |
| Male | 1.11 | [0.98, 1.25] | 0.109 |
| Age |  |  |  |
| Young (<65) | Reference |  |  |
| Old | 1.51 | [1.33, 1.72] | <0.0001 |
| Ferritin |  |  |  |
| Normal | Reference |  |  |
| High (Male>336 or female>306 | 1.53 | [1.35, 1.73] | <0.0001 |
| GEP70 |  |  |  |
| Low (<0.66) | Reference |  |  |
| High | 1.67 | [1.36, 2.05] | <0.0001 |
| GEP80 |  |  |  |
| Low (<2.48) | Reference |  |  |
| High | 1.11 | [0.89, 1.39] | 0.356 |
| PI |  |  |  |
| Low (<10) | Reference |  |  |
| High | 1.17 | [0.92, 1.48] | 0.209 |
| SKY92 |  |  |  |
| Low (<0.827) | Reference |  |  |
| High | 1.51 | [1.27, 1.79] | <0.0001 |

Abbreviation. GEP, gene expression profiling; PI, proliferation index; SKY, skyline.

## Supplementary table6. Multivariable Cox of ferritin and GEP scores (Overall survival)

| **Variables** | **HR** | **95%CI** | **P value** |
| --- | --- | --- | --- |
| Gender |  |  |  |
| Female | Reference |  |  |
| Male | 1.1 | [0.96 ,1.26] | 0.156 |
| Age |  |  |  |
| Young (<65) | Reference |  |  |
| Old | 1.87 | [1.63 ,2.14] | <0.0001 |
| Ferritin |  |  |  |
| Normal | Reference |  |  |
| High (Male>336 or female>306 | 1.68 | [1.48 ,1.92] | <0.0001 |
| GEP70 |  |  |  |
| Low (<0.66) | Reference |  |  |
| High | 1.72 | [1.38 ,2.14] | <0.0001 |
| GEP80 |  |  |  |
| Low (<2.48) | Reference |  |  |
| High | 1.31 | [1.03 ,1.69] | 0.031 |
| PI |  |  |  |
| Low (<10) | Reference |  |  |
| High | 1.12 | [0.88 ,1.41] | 0.364 |
| Sky92 |  |  |  |
| Low (<0.827) | Reference |  |  |
| High | 1.69 | [1.41 ,2.03] | <0.0001 |

Abbreviation. ISS, the International Stage System; ALB, Albumin; B2M, Beta-2 Microglobulin; LDH, Lactate Dehydrogenase.

## Supplementary table7 Multivariable Cox regressions of progression-free survival on demographic and serum-index variables in the 3329-MM subset

| **Variables** | **HR** | **95%CI** | **p val** |
| --- | --- | --- | --- |
| Gender |  |  |  |
| Female | reference |  |  |
| Male | 1.10 | [1.01, 1.20] | 0.028 |
| Age |  |  |  |
| Young (<65) | reference |  |  |
| Old | 1.42 | [1.30, 1.55] | <0.0001 |
| ISS stage |  |  |  |
| ISS I | reference |  |  |
| ISS II | 0.95 | [0.82, 1.09] | 0.458 |
| ISS III | 1.23 | [1.00, 1.51] | 0.053 |
| Ferritin level |  |  |  |
| Normal | reference |  |  |
| High (female>306, male>336) | 1.58 | [1.45, 1.73] | <0.0001 |
| ALB |  |  |  |
| Normal | reference |  |  |
| Low (<3.5) | 1.15 | [1.03, 1.29] | <0.016 |
| B2M |  |  |  |
| Normal (<4) | reference |  |  |
| High | 1.09 | [0.93, 1.27] | 0.290 |
| LDH |  |  |  |
| Normal (<190) | reference |  |  |
| High | 1.26 | [1.15, 1.38] | <0.0001 |
| Creatinine |  |  |  |
| Normal (<2) | reference |  |  |
| High | 0.91 | [0.79, 1.04] | 0.149 |

Abbreviation. ISS, the International Stage System; ALB, Albumin; B2M, Beta-2 Microglobulin; LDH, Lactate Dehydrogenase.

## Supplementary table8. Multivariable Cox regressions of overall survival on demographic and serum-index variables in the 3329-MM subset

| **Variables** | **HR** | **95%CI** | **p Val** |
| --- | --- | --- | --- |
| Gender |  |  |  |
| Female | reference |  |  |
| Male | 1.1 | [1, 1.2] | 0.048 |
| Age |  |  |  |
| Young (<65) | reference |  |  |
| Old | 1.74 | [1.59, 1.92] | <0.0001 |
| ISS stage |  |  |  |
| ISS I | reference |  |  |
| ISS II | 0.92 | [0.78, 1.07] | 0.273 |
| ISS III | 1.19 | [0.96, 1.49] | 0.115 |
| Ferritin level |  |  |  |
| Normal | reference |  |  |
| High (female>306, male>336) | 1.64 | [1.5, 1.8] | <0.0001 |
| ALB |  |  |  |
| Normal | reference |  |  |
| Low (<3.5) | 1.29 | [1.14, 1.45] | <0.0001 |
| B2M |  |  |  |
| Normal (<4) | reference |  |  |
| High | 1.17 | [0.99, 1.39] | 0.061 |
| LDH |  |  |  |
| Normal (<190) | reference |  |  |
| High | 1.38 | [1.26, 1.51] | <0.0001 |
| Creatinine |  |  |  |
| Normal (<2) | reference |  |  |
| High | 1.03 | [0.89, 1.18] | 0.687 |

## Supplementary table9. Percentage of high-ferritin MM patients in ISS-series stages

| **Stage systems** | **I** | **II** | **III** | **III** |
| --- | --- | --- | --- | --- |
| ISS (High ferritin/normal) | 0.26 | 0.40 | 0.63 |  |
| R-ISS (High ferritin/normal) | 0.12 | 0.33 | 0.68 |  |
| R2-ISS (High ferritin/normal) | 0.15 | 0.25 | 0.43 | 0.6 |

## Supplementary table10. Median survival of stages in ISS series systems combine with ferritin

|  | Original Median OS [Q1, Q3] | Ferritin high Median OS [Q1, Q3] | Ferritin normal Median OS [Q1, Q3] | Original Median PFS [Q1, Q3] | Ferritin high Median PFS [Q1, Q3] | Ferritin normal Median PFS [Q1, Q3] |
| --- | --- | --- | --- | --- | --- | --- |
| ISS stage I | 106.6 [60.5, 160.2] | 87.7 [36.9, 143.8] | 110.3 [67.5, 160.4] | 75.4 [36.4, 132.2] | 51.7 [23.7, 98.8] | 80.6 [42.3, 135.5] |
| ISS stage II | 85.6 [49.4, 132.5] | 74.5 [37.5, 119.5] | 91.6 [56.0, 140.1] | 63.3 [26.4, 112.0] | 45.4 [16.8, 94.5] | 69.7 [32.8, 118.2] |
| ISS stage III | 65.7 [28.4, 114.9] | 52.9 [21.7, 102.6] | 83.5 [42.4, 127.8] | 44.6 [17.6, 91.1] | 14.5 [14.5, 79.9] | 58.4 [24.5, 105.8] |
| R-ISS stage I | 107.0 [78.0, 141.4] | 108.5 [80.0, 172.0] | 107.1 [77.9, 135.6] | 84.2 [45.9, 125.6] | 86.2 [37.1, 144.8] | 83.4 [47.5, 124.9] |
| R-ISS stage II | 89.9 [50.4, 136.6] | 76.1 [38.4, 125.6] | 95.2 [57.5, 142.3] | 67.7 [29.4, 118.4] | 56.2 [21.1, 100.0] | 72.8 [35.3, 123.9] |
| R-ISS stage III | 52.6 [22.6, 103.4] | 38.9 [15.8, 84.3] | 93.1 [42.6, 131.0] | 33.9 [13.2, 77.0] | 25.0 [9.6, 58.3] | 56.4 [27.7, 99.2] |
| R2-ISS stage I | 109.4 [80.0, 143.5] | 114.3 [76.9, 160.2] | 109..4 [80.9, 142.0] | 89.3 [52.7, 128.1] | 90.3 [54.5, 154.0] | 88.0 [52.6, 126.5] |
| R2-ISS stage II | 100.8 [66.2, 141.0] | 100.1 [69.3, 148.5] | 100.9 [66.5, 140.1] | 73.8 [38.1, 124.9] | 81.8 [36.0, 115.8] | 71.4 [41.6, 125.3] |
| R2-ISS stage III | 82.4 [42.0, 126.0] | 64.0 [28.7, 108.5] | 92.4 [53.8, 133.1] | 54.5 [22.2, 95.1] | 36.7 [15.4, 78.2] | 64.0 [29.7, 110.9] |
| R2-ISS stage IV | 42.1 [21.6, 84.9] | 32.5 [16.6, 65.1] | 63.7 [22.4, 101.8] | 27.6 [11.5, 52.6] | 27.0 [11.5, 45.1] | 32.0 [16.0, 76.5] |

## Supplementary table11 Baseline of 708 non-transplant MM patients

| **Variables** | **Total (n = 708)** | **high (n = 267)** | **normal (n = 441)** | **p** |
| --- | --- | --- | --- | --- |
| Sex, n (%) |  |  |  | 0.719 |
| Female | 323 (46) | 119 (45) | 204 (46) |  |
| Male | 385 (54) | 148 (55) | 237 (54) |  |
| age, Median (Q1,Q3) | 65.2 (57.04, 72.51) | 66.22 (57.19, 72.29) | 64.64 (56.96, 72.97) | 0.572 |
| Race, n (%) |  |  |  | 0.009 |
| Asian | 5 (1) | 3 (1) | 2 (0) |  |
| Black | 123 (17) | 59 (22) | 64 (15) |  |
| Native American | 2 (0) | 0 (0) | 2 (0) |  |
| White / Caucasian | 555 (78) | 194 (73) | 361 (82) |  |
| NA | 23 (3) | 11 (4) | 12 (3) |  |
| Ferritin, Median (Q1,Q3) | 217.15 (80.07, 531.92) | 736.7 (465.2, 1330.5) | 103.4 (50.7, 195.4) | < 0.001 |
| ISS, n (%) |  |  |  | < 0.001 |
| I | 250 (35) | 36 (13) | 214 (49) |  |
| II | 214 (30) | 76 (28) | 138 (31) |  |
| III | 240 (34) | 153 (57) | 87 (20) |  |
| NA | 4 (1) | 2 (1) | 2 (0) |  |
| PFS, n (%) |  |  |  | < 0.001 |
| No progression/Missing | 238 (34) | 56 (21) | 182 (41) |  |
| Progression | 470 (66) | 211 (79) | 259 (59) |  |
| PFS TIME, Median (Q1,Q3) | 32.78 (9.67, 65.3) | 24.77 (5.53, 55.3) | 39.33 (11.53, 73.5) | < 0.001 |
| OS, n (%) |  |  |  | < 0.001 |
| Alive/Missing | 290 (41) | 68 (25) | 222 (50) |  |
| Death | 418 (59) | 199 (75) | 219 (50) |  |
| OS TIME, Median (Q1,Q3) | 46.65 (15.69, 81.63) | 36.7 (12.7, 70.22) | 52.1 (19.47, 93.03) | < 0.001 |

Q1, the first quartile; Q3, the third quartile.

# Reference

1. Zhan F, Hardin J, Kordsmeier B, Bumm K, Zheng M, Tian E*, et al.* Global gene expression profiling of multiple myeloma, monoclonal gammopathy of undetermined significance, and normal bone marrow plasma cells. *Blood* 2002 Mar 1; **99**(5)**:** 1745-1757.

2. Zhan F, Huang Y, Colla S, Stewart JP, Hanamura I, Gupta S*, et al.* The molecular classification of multiple myeloma. *Blood* 2006 Sep 15; **108**(6)**:** 2020-2028.

3. Shaughnessy JD, Jr., Zhan F, Burington BE, Huang Y, Colla S, Hanamura I*, et al.* A validated gene expression model of high-risk multiple myeloma is defined by deregulated expression of genes mapping to chromosome 1. *Blood* 2007 Mar 15; **109**(6)**:** 2276-2284.

4. Shaughnessy JD, Jr., Qu P, Usmani S, Heuck CJ, Zhang Q, Zhou Y*, et al.* Pharmacogenomics of bortezomib test-dosing identifies hyperexpression of proteasome genes, especially PSMD4, as novel high-risk feature in myeloma treated with Total Therapy 3. *Blood* 2011 Sep 29; **118**(13)**:** 3512-3524.

5. Kuiper R, Broyl A, de Knegt Y, van Vliet MH, van Beers EH, van der Holt B*, et al.* A gene expression signature for high-risk multiple myeloma. *Leukemia* 2012 Nov; **26**(11)**:** 2406-2413.

6. Hanamura I, Stewart JP, Huang Y, Zhan F, Santra M, Sawyer JR*, et al.* Frequent gain of chromosome band 1q21 in plasma-cell dyscrasias detected by fluorescence in situ hybridization: incidence increases from MGUS to relapsed myeloma and is related to prognosis and disease progression following tandem stem-cell transplantation. *Blood* 2006 Sep 1; **108**(5)**:** 1724-1732.

7. Shaughnessy J, Jacobson J, Sawyer J, McCoy J, Fassas A, Zhan F*, et al.* Continuous absence of metaphase-defined cytogenetic abnormalities, especially of chromosome 13 and hypodiploidy, ensures long-term survival in multiple myeloma treated with Total Therapy I: interpretation in the context of global gene expression. *Blood* 2003 May 15; **101**(10)**:** 3849-3856.

8. Xiong W, Wu X, Starnes S, Johnson SK, Haessler J, Wang S*, et al.* An analysis of the clinical and biologic significance of TP53 loss and the identification of potential novel transcriptional targets of TP53 in multiple myeloma. *Blood* 2008 Nov 15; **112**(10)**:** 4235-4246.

9. Chang H, Qi C, Yi QL, Reece D, Stewart AK. p53 gene deletion detected by fluorescence in situ hybridization is an adverse prognostic factor for patients with multiple myeloma following autologous stem cell transplantation. *Blood* 2005 Jan 1; **105**(1)**:** 358-360.

10. Durie BG, Harousseau JL, Miguel JS, Blade J, Barlogie B, Anderson K*, et al.* International uniform response criteria for multiple myeloma. *Leukemia* 2006 Sep; **20**(9)**:** 1467-1473.

11. Greipp PR, San Miguel J, Durie BG, Crowley JJ, Barlogie B, Blade J*, et al.* International staging system for multiple myeloma. *Journal of clinical oncology : official journal of the American Society of Clinical Oncology* 2005 May 20; **23**(15)**:** 3412-3420.

12. Palumbo A, Avet-Loiseau H, Oliva S, Lokhorst HM, Goldschmidt H, Rosinol L*, et al.* Revised International Staging System for Multiple Myeloma: A Report From International Myeloma Working Group. *Journal of clinical oncology : official journal of the American Society of Clinical Oncology* 2015 Sep 10; **33**(26)**:** 2863-2869.

13. D'Agostino M, Cairns DA, Lahuerta JJ, Wester R, Bertsch U, Waage A*, et al.* Second Revision of the International Staging System (R2-ISS) for Overall Survival in Multiple Myeloma: A European Myeloma Network (EMN) Report Within the HARMONY Project. *Journal of clinical oncology : official journal of the American Society of Clinical Oncology* 2022 Oct 10; **40**(29)**:** 3406-3418.

14. Kaplan EL, Meier P. Nonparametric-Estimation from Incomplete Observations. *J Am Stat Assoc* 1958; **53**(282)**:** 457-481.

15. Peto R, Peto J. Asymptotically Efficient Rank Invariant Test Procedures. *J R Stat Soc Ser a-G* 1972; **135:** 185-&.

16. Cox DR. Regression Models and Life-Tables. *J R Stat Soc B* 1972; **34**(2)**:** 187-+.

17. Zhang Z, Gayle AA, Wang J, Zhang H, Cardinal-Fernandez P. Comparing baseline characteristics between groups: an introduction to the CBCgrps package. *Ann Transl Med* 2017 Dec; **5**(24)**:** 484.
